# Structural and morphological data of RF-Sputtered BiVO_4_ thin films

**DOI:** 10.1016/j.dib.2018.01.070

**Published:** 2018-02-02

**Authors:** R. Venkatesan, S. Velumani, K. Ordon, M. Makowska-Janusik, G. Corbel, A. Kassiba

**Affiliations:** a Department of Electrical Engineering (SEES), CINVESTAV-IPN, Zacatenco, Av IPN #2508, Col Zacatenco, D.F. C.P. 07360, Mexico; b Institute of Molecular and Materials of Le Mans – UMR-CNRS 6283, Le Mans University, 70285 Le Mans, Lns France; c Institute of Physics, Jan Dlugosz University in Czestochowa, Al.Armii Krajowej, 13/15, 42 200 Czestochowa, Poland

## Abstract

Structural and morphological modulation of rf-sputtered BiVO_4_ thin films deposited using mechanochemical synthesis prepared BiVO_4_ nano-powders as sintered target are included in this data article. The crystalline nature of as-prepared films, namely amorphous and crystalline was acquired with time and temperature dependent in-situ high temperature X-ray diffraction (HT-XRD), at a time interval of 1 h. Typical Fourier transform infrared (FT-IR) spectra of annealed thin film of monoclinic BiVO_4_ structure is given. Furthermore, correlation between morphologies of various substrate temperature fabricated BiVO_4_ thin films are presented.

**Specifications Table**

**Table:** 

Subject area	*Physics, Material science, Chemistry, Physical chemistry*
More specific subject area	Materials physics, photocatalysis.
Type of data	*Graphs (HT-XRD and FT-IR) and images (FE-SEM).*
How data was acquired	PANalytical X-ray diffractometer (HT-XRD)*,* Nicolet (Thermo scientific) 510 FT-IR spectrometer, Carl Zeiss Auriga FE-SEM.
Data format	*Analyzed.*
Experimental factors	*In-situ HT-XRD investigations under air atmosphere at variable temperatures.*
Experimental features	*Formation of visible light active monoclinic phase of BiVO*_*4*_*is realized.*
	*Variations with substrate temperature show the notable changes in surface morphology.*
Data source location	*Universite du Maine, Le Mans, France. Cinvestav-IPN, Mexico D.F.*
Data accessibility	*The data are provided with this article.*

**Value of the data**

● Combination methods (mechanochemical and rf-sputtering) can be used to for thin film preparations.
● Similar experimental parameters were adopted to prepare BiVO_4_ thin films on silicon, borosilicate and glass substrate using rf-sputtering and HT-XRD data presented in Fig. S1 provide the formation of visible active narrow band gap (2.4 eV) monoclinic BiVO_4_ structure on glass and silicon substrate.
● The FE-SEM images provide a useful point that by changing substrate temperature one can tune the morphology of BiVO_4_ thin films for desired applications.
● The data is useful to design for structural and morphological dependent device application, including photocatalysis, photo-electrochemical, solar cell, transparent semiconductor fabrications.

## 1 Data

Pristine BiVO_4_ was coated on silicon, borosilicate and glass substrates at various substrate temperature under inert (argon) atmosphere by rf-sputtering method with 50 W power onto a 3.3 cm BiVO_4_ target diameter (mechanochemically prepared [1]). X-ray diffraction characterization was applied to clarify the crystalline nature of the as-deposited films. In-situ HT-XRD thermal treatment was applied to ensure the formation of monoclinic crystalline phase of BiVO_4_. So, the data set used in this data in brief article contains the structural (HT-XRD and FT-IR) data of the room temperature sputtered BiVO_4_ films along with morphological features of various substrate temperature deposited films. Furthermore, the data furnished here are based on the additional experimental observation reported in our recent paper [2]. Graph on the chemical composition, structural changes and characteristics of BiVO_4_ thin films are presented in Fig. S1. HT-X-ray diffraction patterns indicates that the as-deposited films are amorphous in nature at room temperature. The order of the crystallinity increases from amorphous to monoclinic phases of BiVO_4_ with increasing the heating temperature (from RT to 410 °C). Typical monoclinic crystalline nature of BiVO_4_ are generated for thin film deposited on Si substrate at room temperature and annealed at 400 °C (Fig. S2). Fig. S3 provides the morphological comparison of room temperature deposited films on glass and silicon substrates. Also, images showing the effect of substrate temperature on surface morphology of BiVO_4_ films deposited on Si substrate was gathered and depicted in Fig. S4.

## 2 Experimental design, materials, and methods

The crystalline structure of the as-deposited thin films was characterized by PANalytical X-ray diffractometer with Cu Kα radiation (*λ* = 0.15406 nm) equipped with the X’celerator detector and a HTK 1200 Anton Paar chamber. FT-IR measurements were performed by Nicolet 510 spectrometer. Field emission scanning electron microscopy (FE-SEM) was conducted using Carl Zeiss Auriga 60, nanotechnology system equipped with an energy dispersive spectrometer at an accelerating voltage of 2 kV.
