# Supplementary material for: Structural and morphological data of RF-Sputtered BiVO4 thin films
Source: Data Brief. 2018 Feb 2;17:526–8. doi: 10.1016/j.dib.2018.01.070 (PMC5988375; doi:10.1016/j.dib.2018.01.070)
Supplement: Supplementary file 1 — Transparency document [file mmc1.docx]

**Conflict of Interest**

The authors R.Venkatesan ^(1,2)^, S.Velumani ^(1)^, K.Ordon ^(2,3)^ , M.Makowska-Janusik ^(3)^, G.Corbel ^(2)^ and A.Kassiba of the DIB contribution Structural and morphological data of RF-Sputtered BiVO_4_ thin films, declare that there is no conflict of interest of any kind related to the submitted work.
