# Supplementary material for: Structural and morphological data of RF-Sputtered BiVO4 thin films
Source: Data Brief. 2018 Feb 2;17:526–8. doi: 10.1016/j.dib.2018.01.070 (PMC5988375; doi:10.1016/j.dib.2018.01.070)
Supplement: Supplementary file 2 — Supplementary material [file mmc2.docx]

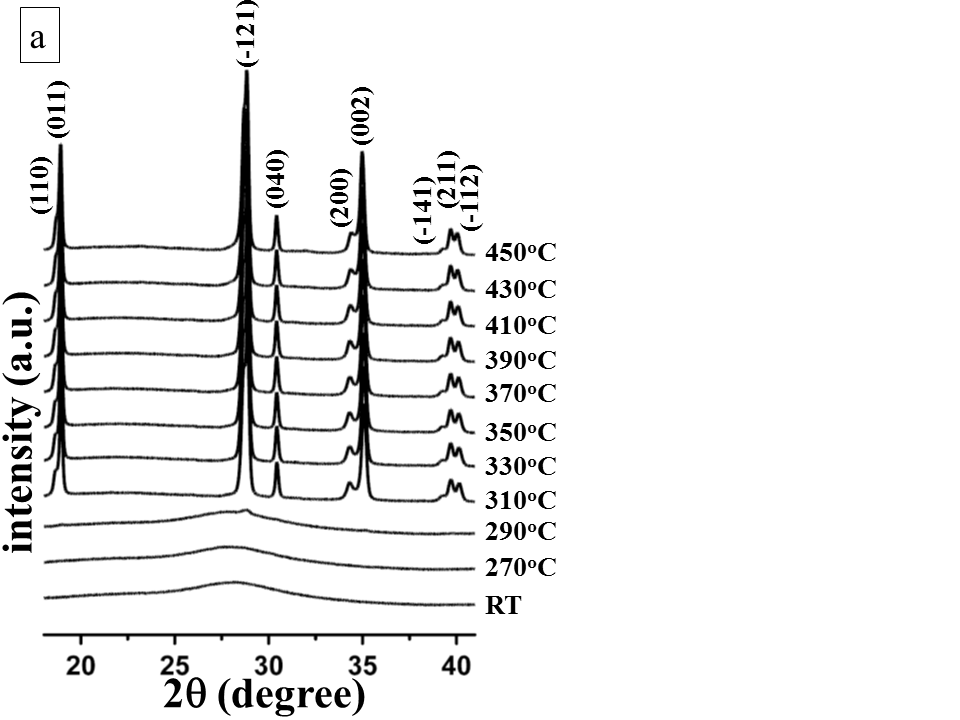

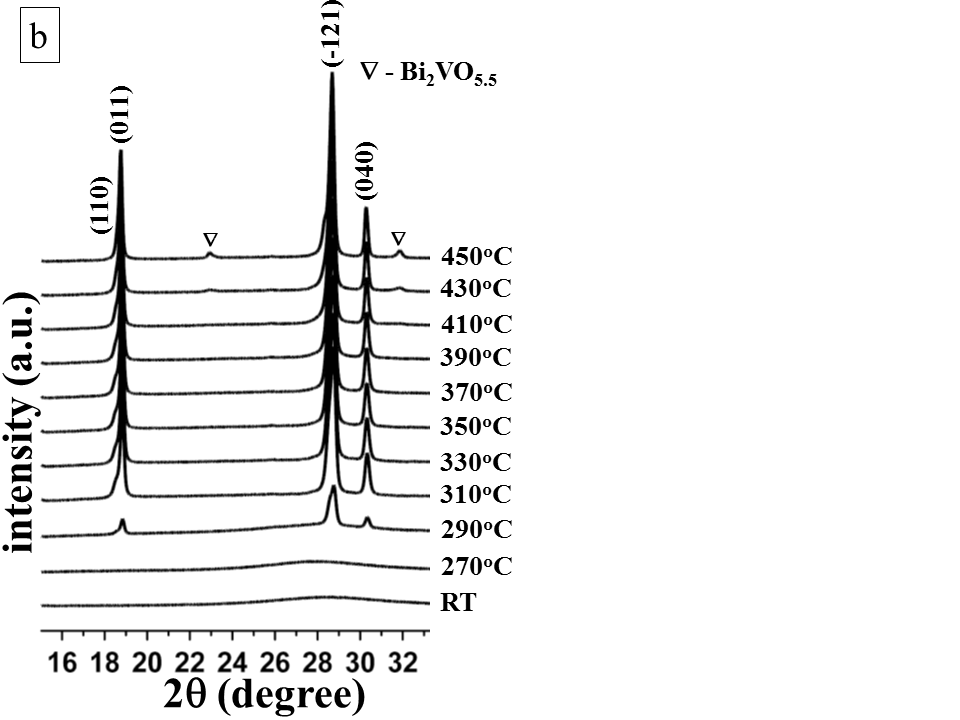


**Figure S1.** In – situ HT-XRD patterns of BiVO_4_ film deposited at room temperature and annealed in-situ under air atmosphere. a) Glass substrate, b) Si Substrate.


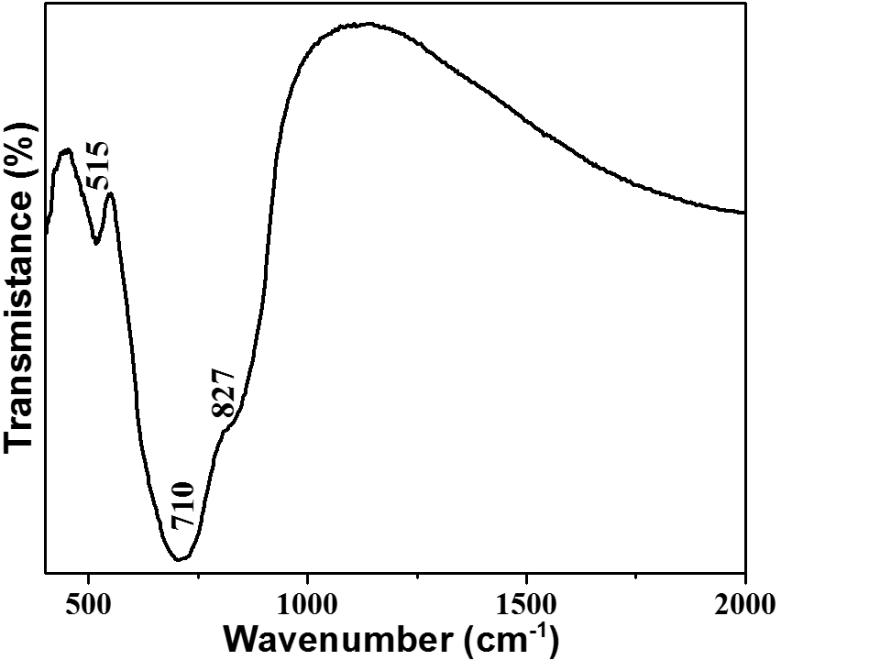


**Figure S2.** FT-IR spectra of BiVO_4_ thin film deposited on Si substrate at RT and annealed at 400^o^C.


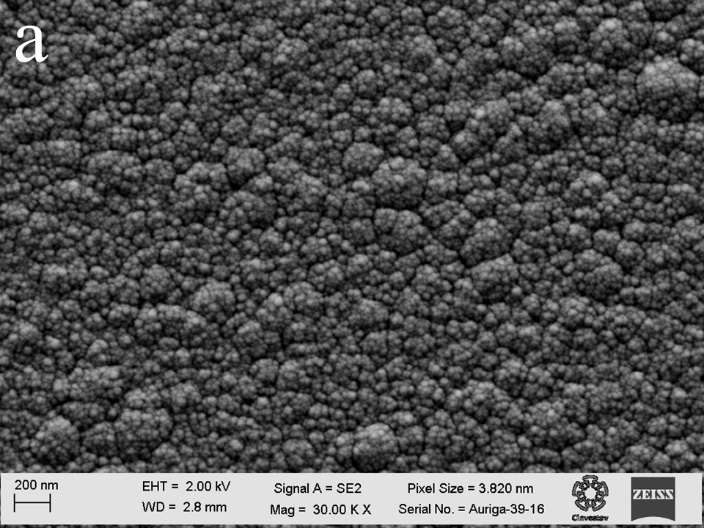

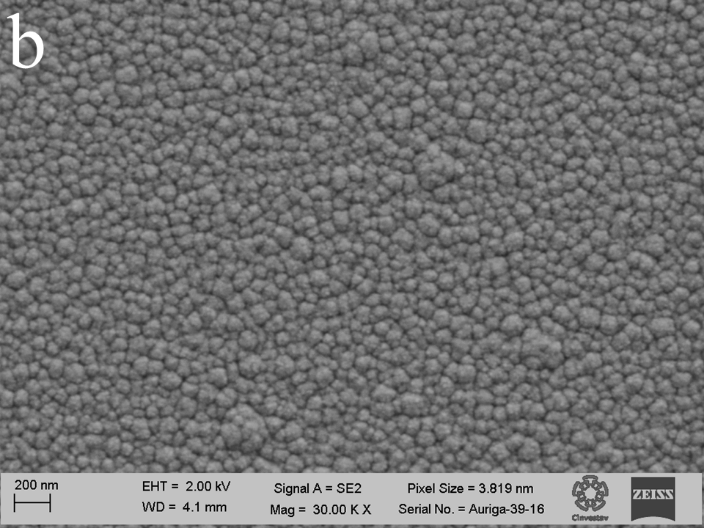


**Figure S3.** FE-SEM images of BiVO_4_ thin films deposited at RT. a) Glass substrate, b) Si substrate.


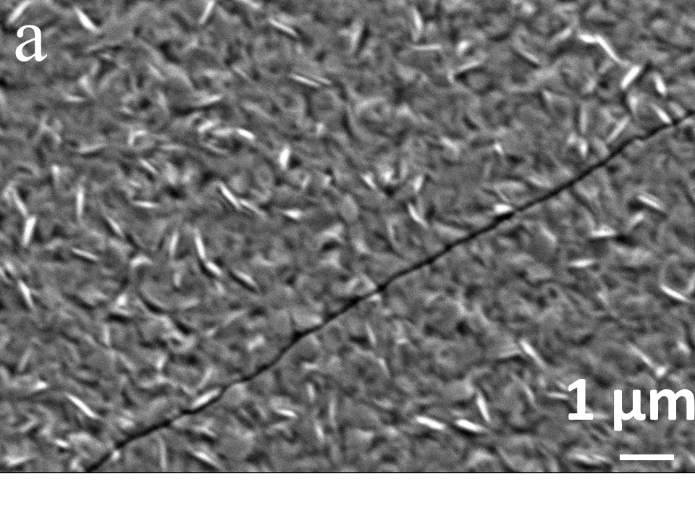

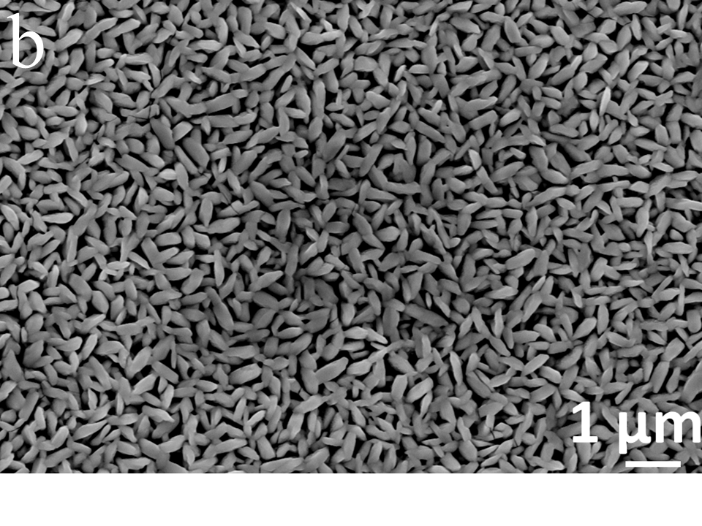


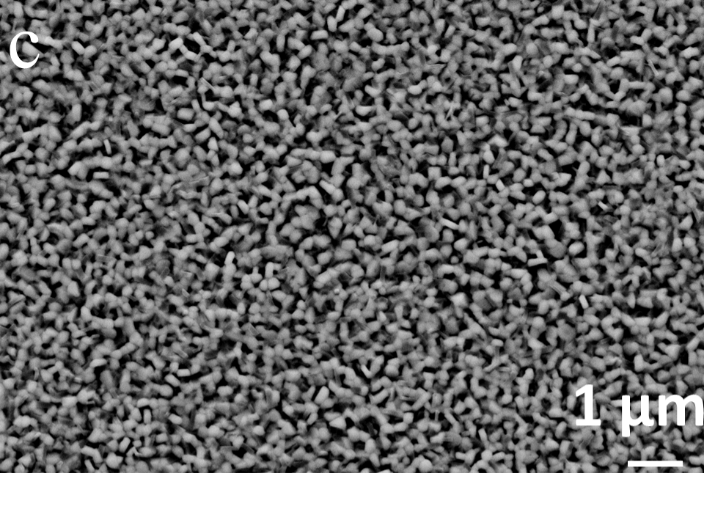


**Figure S4.** FE-SEM images of BiVO_4_ thin films deposited on Si substrate at different substrate temperature. a) 450^o^C, b) 550^o^C, c) 600^o^C.

**Acknowledgements**

We acknowledge the financial support from European Union FP7 – NMP EU- Mexico program under grant agreement n^o^ 263878/ by CONACYT n^o^ 125141. The work has also benefited from financial support of the National Science Centre (Poland) within the framework of the project 2016/21/N/ST3/00455. Mr.Josue at LANE, Cinvestav for the AFM facility. Venkatesan Rajalingam is also thankful for the scholarship jointly provided by SEP and CINVESTAV as well as the joint funding provided by Cinvestav- Le Mans University.

**References**

[1] R. Venkatesan, S. Velumani, A. Kassiba, Mechanochemical synthesis of nanostructured BiVO4 and investigations of related features, Materials Chemistry and Physics, 135 (2012) 842-848.

[2] R. Venkatesan, S. Velumani, K. Ordon, M. Makowska-Janusik, G. Corbel, A. Kassiba, Nanostructured bismuth vanadate (BiVO4) thin films for efficient visible light photocatalysis, Materials Chemistry and Physics, 205 (2018) 325-333.
